# Decomposing socioeconomic inequalities in depressive symptoms among the elderly in China

**DOI:** 10.1186/s12889-016-3876-1

**Published:** 2016-12-01

**Authors:** Yongjian Xu, Jinjuan Yang, Jianmin Gao, Zhongliang Zhou, Tao Zhang, Jianping Ren, Yanli Li, Yuyan Qian, Sha Lai, Gang Chen

**Affiliations:** 1 School of Public Policy and Administration, Xi’an Jiaotong University, No.28, Xianning West Road, Xi’an, Shaanxi China; 2 School of Public Health, Health Science Center, Xi’an Jiaotong University, Xi’an, China; 3 School of Medicine, Hangzhou Normal University, Hangzhou, China; 4 Centre for Health Economics, Monash Business School, Monash University, Melbourne, Australia

**Keywords:** Depressive symptoms, Health inequality, Concentration index, Decomposition analysis

## Abstract

**Background:**

Accelerated population ageing brings about unprecedented challenges to the health system in China. This study aimed to measure the prevalence and the income-related inequality of depressive symptoms, and also identify the determinants of depressive symptom inequality among the elderly in China.

**Methods:**

Data were drawn from the second wave of the China Health and Retirement Longitudinal Study (CHARLS). Depressive symptoms were assessed with a 10-item Center for Epidemiologic Studies–Depression Scale (CES-D), which was preselected in CHARLS. The concentration index was used to measure the magnitude of income-related inequality in depressive symptoms. A decomposition analysis, based on the logit model, was employed to quantify the contribution of each determinant to total inequality.

**Results:**

More than 32.55% of the elderly in China had depressive symptoms. Women had a higher prevalence of depressive symptoms than men. The overall concentration index of depressive symptoms was -0.0645 among the elderly, indicating that depressive symptoms are more concentrated among the elderly who lived in economically disadvantaged situations, favoring the rich. Income was found to have the largest percentage of contribution to overall inequality, followed by residents’ location and educational attainment.

**Conclusion:**

The prevalence of depressive symptoms in the elderly was considerably high in China. There was also a pro-rich inequality in depressive symptoms amongst elderly Chinese. It is suggested that some form of policy and intervention strategies, such as establishing the urban-rural integrated medical insurance scheme, enhancing the medical assistance system, and promoting health education programs, is required to alleviate inequitable distribution of depressive symptoms.

**Electronic supplementary material:**

The online version of this article (doi:10.1186/s12889-016-3876-1) contains supplementary material, which is available to authorized users.

## Background

With large numbers of the population bulge moving into older age, combined with continued low fertility owing to the one-child policy and low elderly mortality, ageing is rapidly accelerating in China. The percentage of people aged 60 years and over has jumped from 10.5% in 2000 to 15.5% in 2015, and is expected to reach 34.5% by 2050 [1, 2]. The life expectancy at birth in China was 74 for men and 77 for women in 2013, while these figures were just 66.9 for men and 70.5 for women in 1980 [3]. Although people are living longer than ever before, the health status of the elderly in China is not encouraging [4]. Population ageing brings about unprecedented challenges to the overburdened health system in China.

Depression, which is one of the most common mental disorders, can cause great suffering, impaired functioning in daily life, decreased health-related quality of life, and even suicide. The World Health Organization (WHO) reported that unipolar depression occurred in 7% of the elderly population [5]. In China, it is estimated that nearly 30% of men and 43% of women aged 45 and over suffered from depressive symptoms in 2011 [6]. Although many effective interventions can be used to alleviate symptoms of depression, symptoms in the elderly are often overlooked and untreated, in that most of them mistakenly deem depression to be part of the normal ageing process. Since China has the largest number of elderly in the world, the challenges of depressive symptoms in the elderly needs to be recognized in China.

In addition to the high prevalence of depressive symptoms, the issue of whether these symptoms are distributed equally among people within different economic status also needs much more attention. There is increasing recognition of a negative association between income inequality and the health status of a population [7, 8]. The potential mechanism is that inadequate spending on health among the poor hinders them from accessing proper health services [9]. Higher inequality also erodes social cohesion and capital and increases chronic stress, which in turn results in poor health outcomes [10]. Furthermore, invidious social comparisons bred by income inequality can cause a variety of psychosocial and physiological problems.

The concentration index has been widely used for measuring income-related inequalities in the health sector internationally, whilst its decomposition analysis has been increasingly adopted to study inequality determinants [11–13]. The majority of previous studies investigated health utilization inequality in China, with very limited study focused on health status [12, 14]. So far, no study has been published to explore inequalities in depressive symptoms among the elderly in China.

The present study has three objectives: firstly, to investigate the prevalence of depressive symptoms among the elderly in China; secondly, to measure the income-related inequality in the probability of suffering from depressive symptoms amongst the elderly; lastly, to quantify each determinants’ contribution to total inequalities. The results will help identify the vulnerable elderly who are mostly affected by depressive symptoms in China, such that they can be targeted by future policy interventions. In addition, the results of this study will shed light on relevant health policy and public health interventions on how to reduce income-related inequality in developing countries.

## Methods

### Data

This study used the data from the second wave of the China Health and Retirement Longitudinal Study (CHARLS). The CHARLS, which is conducted every two years, is a nationally representative survey of middle-aged and older adults and their spouses in China. The second wave of CHARLS was conducted between July 2013 and January 2014, involving 18,605 respondents. A detailed description of the sampling method, quality assurance measures, and the questionnaire has been previously published [15, 16]. The CHARLS data can be accessed through its official website (charls.ccer.edu.cn/en).

In this study, a sub-sample of respondents aged ≥60 years old and who had self-completed the measure of depressive symptoms was used (*n* = 7579). After data cleaning (i.e. excluding elderly respondents with logic error answers or with key variables missing), 6,522 respondents were adopted for this study.

### Outcome variable

Depressive symptoms were assessed utilizing a Chinese version 10-item short form of the Center for Epidemiologic Studies–Depression Scale (CES-D) which was preselected in CHARLS. The questions of 10-item CES-D scale are presented in Additional file 1. A previous study has demonstrated that the Chinese version of the 10-item CES-D has exhibited a good internal consistent reliability in the elderly population, with a Cronbach’s alpha is 0.813 [16]. Several studies have also demonstrated the good construct and factorial validity of Chinese version of CES-D scale [16, 17].

The 10-item CES-D questionnaire consists of eight negatively oriented and two positively oriented items, e.g. “I felt everything I did was an effort” or “I was happy”. Each item has 4 response levels, ranging from rarely or none of the time (<1 day), some or a little of the time (1–2 days), occasionally or a moderate amount of the time (3–4 days), to most or all of the time (5–7 days). Eight negatively oriented items were scored from zero for rarely or none of the time to three for most of the time, whereas the remaining two positively oriented items were scored in reverse. The 10 items were summed to obtain a total CES-D score for a respondent which ranged from 1 to 30. Previous studies have recommended that the cut-off point for depressive symptoms in older adults is 10 for the 10-item CES-D scale [18]. A score of 10 and over was used as the measure of depressive symptoms in the study.

### Independent variables

Numerous variables are available in CHARLS. Followed by prior empirical investigations, all possible variables that may produce mental health problems were initially considered [19–21]. After eliminating redundant variables, four groups of variables were included in our study. Demographic characteristics considered in the study were age (60–64, 65–74, 75 years and older), sex, and living arrangement (living alone, living with their spouse, other arrangement). Socioeconomic characteristics considered in the study were income, educational attainment (illiterate, capable of reading or writing, elementary school, middle school and above), and working status. Income was measured by per capita net household expenditure. Net household expenditure was calculated as total household consumption expenditure minus household health expenditure [22]. A set of location dummies was included to capture the potential regional heterogeneity. Geographic characteristics consisted of six urban-rural and location (east, central, and west) categories, with “urban-east” as the reference group. Life-style and health behavior variables considered in the study were social activity and smoking. Social activity was measured by the question, “Have you done any of these activities in the last month?” Possible answers to this question were ten informal social interactions (e.g. interacted with friends, took part in a community-related organization), among which seven activities were considered as social activities. A social activity variable was created to count the number of social activities that respondents participated in during the last month. Lastly, physical disabilities, brain damage, vision problems, hearing problems were established to indicate whether respondents had corresponding disabilities.

### Statistical analysis

Concentration curve and concentration index (C) were employed to depict the inequalities in the distribution of depressive symptoms by income. The concentration curve gives a complete picture of how shares of the health variable (y-axis) are accounted for by the cumulative percentage of individuals ranked by living standards from the poorest to the richest (x-axis). If an individual, irrespective of his or her income, has an identical health outcome, the curve will be a 45° line, which runs from the lower left corner to the upper right corner (also known as the line of equality). If the health outcomes measure takes higher values among poorer people, the concentration curve will lie above the line of equality, and vice versa. The concentration index is a standard tool for measuring the magnitude of income-related inequality, and is defined as twice the area between the concentration curve and the line of equality. The concentration index is bound between -1 and +1. Its negative values imply that health variables are more concentrated among the poor, and vice versa. If there is no income-related inequality, the concentration index is zero. Details on how to compute the concentration index and corresponding standard error can be found in previous studies [23, 24].

Decomposition analysis was first used in the field of economics, and then gradually adopted in public health and epidemiology to decompose the concentration index into the contribution of various factors. If the health outcome is a continuous variable, the decomposition approach based on OLS regression was commonly adopted [25]. The linear relationship between health outcome (y_i_) and independent variable (x_k_) can be written as:

$$ {y}_i=\alpha +{\displaystyle {\sum}_k{\beta}_k{x}_{ki}+{\varepsilon}_i} $$

The concentration index for y, can be written as:

$$ c={\displaystyle {\sum}_k\left({\beta}_k\overline{x_k}/\mu \right)\;}{c}_k+G{C}_{\varepsilon }/\mu $$

Where *c* denotes the overall concentration index, *β*_*k*_ and $\overline{x_k}$ are regressors and the mean of *x*_*k*_, respectively, *c*_*k*_ is the concentration index of *x*_*k*_ (defined exactly like C), *GC*_*ε*_ is the generalized concentration index for *ε*. The equation shows that the concentration index is made up of two components: explained component and residual (unexplained) component.

In most cases, however, health outcome variables are seldom continuous. Marginal effect based on logit model can be opted to approximate the decomposition analysis. A linear approximation of the non-liner estimation can be written as:

$$ {y}_i={\alpha}^m+{\displaystyle {\sum}_k{\beta}_k^m}{x}_{ki}+{\mu}_i $$

Where *β*_*k*_^*m*^ is the marginal effects (dy/dx) of each x; *μ*_*i*_ indicates the error term generated by the linear approximation. The concentration index for the interested variable y can be written as:

$$ C={\displaystyle {\sum}_k\left({\beta}_k^m\overline{x_k}/\mu \right){C}_k+\left(\overline{\varepsilon}/\mu \right){C}_{\mu }} $$

## Results

The characteristics of respondents are described in Table 1. A total of 6,522 older adults, including 3,403 (52.18%) men and 3,119 (47.82%) women, participated in our study with the mean (standard deviation, SD) age of 67.57 (6.31) years. More than 60% of respondents lived in rural areas; 54.66% of respondents were still working; 31.87% of respondents were illiterate; and 9.18% of respondents were living alone.

**Table 1:** Characteristics of respondents

Characteristics	All (*N* = 6522)
Age, years	
mean (SD)	67.57 ± 6.311
Sex	
Men, n (%)	3403 (52.18)
Women, n (%)	3119 (47.82)
Living arrangement	
Living alone, n (%)	599 (9.18)
Living with their spouse, n (%)	2702 (41.43)
Other arrangement, n (%)	3221 (49.39)
Income, RMB yuan	
mean (SD)	8306.09 ± 12553.39
Education	
Illiterate, n (%)	2079 (31.87)
Capable of reading or writing, n (%)	1368 (20.97)
Elementary school, n (%)	1646 (25.24)
Middle school and above, n (%)	1429 (21.91)
Working status	
Yes, n (%)	3565 (54.66)
No, n (%)	2957 (45.34)
Residential location	
Eastern China, n (%)	2184 (33.49)
Central China, n (%)	2182 (33.46)
Western China, n (%)	2156 (33.06)
Residential areas	
Urban, n (%)	2583 (39.60)
Rural, n (%)	3939 (60.4)
Social activity	
mean (SD)	0.74 ± 0.863
Smoking	
Yes, n (%)	3042 (46.64)
No, n (%)	3480 (53.36)
Physical disabilities	
Yes, n (%)	6093 (93.42)
No, n (%)	429 (6.58)
Brain damage	
Yes, n (%)	6198 (95.03)
No, n (%)	324 (4.97)
Vision problems	
Yes, n (%)	5742 (88.04)
No, n (%)	780 (11.96)
Hearing problems	
Yes, n (%)	5439 (83.39)
No, n (%)	1083 (16.61)

Note: *SD* standard deviation

Table 2 presents the prevalence of depressive symptoms among the elderly in China. As can be seen, the prevalence of depressive symptoms was 32.55% in the elderly. Women had consistently higher prevalence of depressive symptoms than men among all three age groups. Those living in rural areas also had a consistently higher proportion of depressive symptoms than those living in urban areas in any age group. Women aged 60–64 years, and living in rural areas, had the highest proportion of depressive symptoms, whilst men aged 75 and older, living in urban areas, had the lowest incidence of suffering depression symptoms (47.22% vs. 18.88%).

**Table 2:** Number and proportion of respondents having depressive symptoms by sex and age

	Urban		Rural		Total	
	N	%	N	%	N	%
Men						
60–64	98	19.25	226	28.36	324	24.81
65–74	123	21.73	293	30.02	416	26.98
≥ 75	44	18.88	102	31.68	146	26.31
Women						
60–64	174	32.22	365	47.22	539	41.05
65–74	159	30.40	369	45.17	528	39.40
≥ 75	61	28.77	109	42.91	170	36.48
Total (60+)	659	25.51	1464	37.17	2123	32.55

Table 3 presents the adjusted associations between depressive symptoms and its determinants, based on the logistic regression. It was found that being a woman, living alone, with lower education attainment, physical disabilities, brain damage and vision problems were associated with an increase in the risk of depressive symptoms, whereas other factors, such as being age 65–74, being age 75 and over, income, social activity were associated with a decreased risk of suffering from depressive symptoms (i.e. odds ratios smaller than 1).

**Table 3:** Adjusted association between depressive symptoms and its determinants based on logistic regression

Variable	Odds Ratio	Std. Err.	z	*P* > z	95% Confidence interval	
					Low	High
Age						
60–64	Ref					
65–74	0.85	0.059	−2.29	0.022	0.747	0.977
≥ 75	0.70	0.070	−3.58	<0.001	0.575	0.851
Sex						
Men	Ref					
Women	1.82	0.164	6.64	<0.001	1.524	2.168
Living arrangement						
Living alone	1.41	0.153	3.13	0.002	1.136	1.740
Living with their spouse	0.96	0.065	−0.54	0.588	0.846	1.100
Other arrangement	Ref					
Income	0.91	0.033	−2.73	0.006	0.844	0.973
Education						
Illiterate	1.32	0.182	2.02	0.044	1.008	1.729
Capable of reading or writing	1.41	0.193	2.51	0.012	1.078	1.843
Elementary school	1.11	0.148	0.77	0.439	0.853	1.441
Middle school and above	Ref					
Working status						
Yes	0.86	0.059	−2.18	0.029	0.752	0.985
No	Ref					
Location						
Urban-eastern	Ref					
Rural-eastern	1.52	0.181	3.51	<0.001	1.202	1.918
Urban-central	1.38	0.180	2.45	0.014	1.066	1.779
Rural-central	1.78	0.211	4.9	<0.001	1.416	2.250
Urban-west	1.58	0.206	3.51	<0.001	1.224	2.041
Rural-west	2.87	0.333	9.06	<0.001	2.282	3.598
Social activity	0.86	0.035	−3.82	<0.001	0.791	0.927
Smoking						
Yes	1.17	0.101	1.84	0.065	0.990	1.387
No	Ref					
Physical disabilities						
Yes	1.65	0.195	4.25	<0.001	1.311	2.084
No	Ref					
Brain damage						
Yes	1.83	0.254	4.34	<0.001	1.393	2.401
No	Ref					
Vision problems						
Yes	1.42	0.130	3.83	<0.001	1.187	1.698
No	Ref					
Hearing problems						
Yes	1.11	0.092	1.28	0.202	0.945	1.307
No	Ref					
_cons	1.80	0.695	1.53	0.125	0.848	3.839

**Fig. 1:**
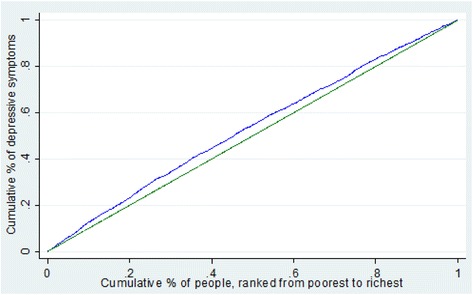
Concentration *curve* of depressive symptoms among elderly in China. The *green line*, running from the *lower left* corner to the *upper right *corner, is the equality line. The *blue line* above the equality line represents the concentration *curve*. The father the concentration *curve* is above the equality line, the more concentrated the ill-health is among the poor

Figure 1 shows the crude concentration index and concentration curve of the probability of suffering from depressive symptoms in the elderly. The concentration curve lay above the 45° line (the line of equality), and the corresponding crude concentration index was -0.0645 (95% confidence interval: -0.08471, -0.04423), indicating that depressive symptoms are more concentrated among the elderly who lived in economically disadvantaged situations, favoring the rich. Total depressive symptoms inequality can be decomposed into two components: “potentially avoidable” inequality and “unavoidable” inequality. By deducting the influence of age-sex expected inequality from the total concentration index, the inequity index (avoidable inequality) became -0.0654.

The elasticities, concentration indices of each exploratory variable, absolute contributions to C, and percentage contributions to C, are presented in Table 4. Concentration indices of each exploratory variable are shown in column 3. Positive values, such as social activity and living alone, means these variables are more concentrated among the rich, and vice versa. The absolute contributions and the percentage of contributions of each variable to the observed inequality are presented in column 4 and column 5. Since the outcome of interest for this study is an indicator of ill health and concentrated among the poor, negative contribution means that these variables increase the degree of pro-rich inequality. That is to say, the poor are more likely to suffer from depressive symptoms than the rich, and vice versa. Decomposition analysis showed that the largest absolute contributions were from income, living in the rural-west areas, living in the rural-central areas, social activity, and being illiterate. These negative contributions are partially offset by positive contributions from living in central and west urban areas and working status. Quantifying the contributions expressed as a percentage of each variable, income, living in the rural-west areas, social activity contributed 51.65%, 23.34%, and 14.48% to the total observed inequality in depressive symptoms.

**Table 4:** Decomposition analysis of concentration index in depressive symptoms among elderly

Variable	Elasticity	C_k_	Absolute contribution to C	Percentage contribution to C
Age				
60–64	Ref			
65–74	−0.046	−0.014	0.001	−1.02
≥ 75	−0.038	−0.087	0.003	−5.11
Sex				
Men	Ref			
Women	0.199	−0.013	−0.003	4.06
Living arrangement				
Living alone	0.023	0.076	0.002	−2.65
Living with their spouse	−0.010	0.080	−0.001	1.23
Other arrangement	Ref			
Income	−0.566	0.059	−0.033	51.65
Education				
Illiterate	0.069	−0.131	−0.009	13.93
Capable of reading or writing	0.058	−0.052	−0.003	4.65
Elementary school	0.021	0.003	0.000	−0.11
Middle school and above	Ref			
Working status				
Yes	−0.057	−0.057	0.003	−5.04
No	Ref			
Location				
Urban-eastern	Ref			
Rural-eastern	0.059	−0.134	−0.008	12.19
Urban-central	0.027	0.143	0.004	−6.02
Rural-central	0.082	−0.124	−0.010	15.88
Urban-west	0.037	0.156	0.006	−8.98
Rural-west	0.170	−0.089	−0.015	23.34
Social activity	−0.073	0.128	−0.009	14.48
Smoking				
Yes	0.048	0.002	0.000	−0.17
No	Ref			
Physical disabilities				
Yes	0.024	−0.052	−0.001	1.93
No	Ref			
Brain damage				
Yes	0.021	0.008	0.000	−0.27
No	Ref			
Vision problems				
Yes	0.031	−0.035	−0.001	1.70
No	Ref			
Hearing problems				
Yes	0.012	−0.029	0.000	0.55
No	Ref			
Residual (unexplained)			−0.075	−16.25

Note: *C*_*k*_ concentration index of explanatory variables, *C* concentration index of independent variable, *Ref* reference group

## Discussion

Depression is a major public health problem that can create a variety of emotional and physical problems. Using the large-scale nationally representative survey data, this study measured the prevalence of depressive symptoms among the elderly in China. The result of this study found that the proportion of older adults with depressive symptoms in China had reached 32.55% in 2013, much higher than a previously reported 14.81% from a meta-analysis on the prevalence of depressive symptoms in the 1980s and early 1990s [26]. Two reasons could partially explain the dramatically increased prevalence of depressive symptoms in the past few decades. First, with the phenomenal economic development in China in the past three decades, the accelerated pace of modern life and increasing pressures of living, coupled with unhealthy life-styles increased the risk of suffering from depressive symptoms. Second, although many health promotion interventions, such as free physical check-ups for elderly people and free operations for poor cataract patients, have been provided by the Chinese government to improve the health status of the elderly since 2009, measures aimed at alleviating depressive disorders were seldom conducted [27]. When left untreated, depression can have serious consequences, affecting every aspect of elderly people’s lives. Therefore, policy interventions aimed at relieving the symptoms of depression in the elderly should be of interest to the Chinese government.

Cross-country comparison showed that the prevalence of depressive symptoms among the elderly in China was also higher than that reported in most studies carried out in other countries and areas [19, 20, 28–30]. However, due to methodological differences, including variation in samples studied, the screening scale used and the cutoff point adopted, the cross-country comparison should be interpreted with caution.

The present study explored the association between a variety of socio-demographic variables and depressive symptoms. Consistent with most previous studies, our study found that increasing income was negatively associated with suffering from depressive symptoms, whereas lower education attainment was positively associated with depressive symptoms. Although a few studies found insignificant association between sex and depressive symptoms, in agreement with most previous studies, we found that women had a higher risk of suffering from depressive symptoms than men [21, 31]. This may in part be owing to the disadvantaged sociocultural roles and psychological attributes related to women’s greater vulnerability to life events and depressive symptoms [32, 33]. The relationship between age and depressive symptoms are inconclusive. Most studies pointed out that age is a risk factor for depressive symptoms. However, unlike most studies, our study found that age was inversely associated with depressive symptoms. Some published studies support our findings. Blazer et al. found that increased age was positively associated with depressive symptoms among the elderly over 65 years old in bivariate analysis; however, after the confounding variables were simultaneously controlled, this relationship was reversed [34]. Geographically, residence in the rural-west, rural-central, rural-east, urban-west and urban-central areas were all associated with increased risk of suffering from depressive symptoms, compared to living in urban-east areas. That may be because living in economically undeveloped areas implies less access to depression treatments and lower social participation. Our findings showed that more than 9% of older adults in China are living alone. Although one previous study conducted in China found that living alone was positively associated with the health status of the elderly, our study found that those living alone had a higher risk of having depressive symptoms [35]. In line with previous studies, our study found that social activity was negatively associated with an increased risk of suffering from depressive symptoms, whereas physical disabilities, brain damage and vision problems elevated the risk of suffering from depressive symptoms [29, 30]. The reason why such disabilities increased the risk of depressive symptoms is that these long-term, severe body disabilities can result in chronic strain, which was demonstrated as being associated with depression. A poor elderly person with residence in rural-west areas, living alone, illiterate, not participating in any social activity, and having physical disabilities, brain damage and vision problems had the highest probability of having symptoms of depression.

A small number of studies have been published on income-related inequality in depressive symptoms internationally [36, 37]. These existing studies retrieved the data from different countries, aimed at different populations, and using different measures of depressive symptoms, so straight comparison should be cautioned. However, these studies, as well as our study, consistently revealed that depressive symptoms are unequally distributed among the income spectrum, with the poor having the higher probability of suffering from depressive symptoms [37].

As expected, most demographic, socioeconomic and geographic characteristic variables showed a positive percentage of contribution to the overall inequality, and the probability of depressive symptoms was more concentrated among the poor. Among them, our findings are in agreement with previous studies, that income made the largest percentage of contribution to pro-rich inequality [36]. Despite income, residence in rural areas (rural-west, rural-central, and rural-east) and being illiterate also exhibited a substantial percentage of contribution to pro-rich inequality. Living in rural areas and being illiterate was more concentrated among the poor elderly. Although being a woman had a higher probability of having depressive symptoms, the contribution of gender to the total inequality was not large. That is because this variable was almost evenly distributed between the advantaged and disadvantaged. The contribution of age, living alone, and working status (i.e. still working) were to reduce the magnitude to which depressive symptoms were concentrated among the worse off. Our findings showed that although statutory retirement age for ordinary workers in China is 60 for men and 55 for women, more than 54% of the elderly still engaged in work due to a variety of reasons, and those people engaged in work are more concentrated among the poor. Regarding life-style variables investigated in this study, social activity also made a substantial contribution to the overall pro-rich inequality in depressive symptoms, and deserves more attention.

Appropriate policy and intervention strategies should be implemented to reduce the income-related inequalities in depressive symptoms. Possible strategies are as follows: firstly, disadvantaged elderly people (lower income, illiterate, etc.) and vulnerable residential areas should be identified. Secondly, narrowing the gap between the rich and the poor through redistributing income measures is a feasible way to reduce pro-rich inequality. Since the compulsory health insurance in China has the redistributive features, optimal health insurance is a feasible way to decrease income inequality. China’s basic health insurance mainly consists of the Urban Employee Basic Medical Insurance (UEBMI) which is designed for urban residents, the Urban Resident Basic Medical Insurance (URBMI) which is designed for unemployed urban residents, and the New Rural Cooperative Medical Insurance (NRCMI) which is designed for the rural population. Elderly residents covered by UEBMI no longer need to pay a premium after they have retired, and have a higher level of insurance protection, while conversely, insured elderly covered by URBMI and NRCMI still need to pay fixed premiums, and have relatively lower levels of insurance protection. Therefore, establishing and improving the urban-rural integrated medical insurance system should be explored. In addition, enhancing the medical assistance system, which renders assistance to depressed people in lower income families or vulnerable areas, can also help reduce pro-rich inequalities. Thirdly, since lower education not only increases the risk of depressive symptoms, but also makes a substantial contribution to income-related inequality, lower education needs to be addressed and given larger investment. This is especially true for elderly women, in that 48.30% of them are illiterate. Education programs on depression for illiterate elderly people should be emphasized. Fourthly, considering large contributions of social activity to pro-rich inequality in depressive symptoms, encouraging poor elderly to engage in all sorts of social activities is of importance to reduce this contribution.

There are some limitations in our study. Firstly, because of the cross-sectional design of our study, causal conclusions cannot be reached. Secondly, all of the data employed in our study were self-reported. Thirdly, recall bias, which can affect the study results, may exist in our study. To limit recall bias, different recall periods were used in CHARLS, i.e. one week was used for food expenditure, one month for communication, local transportation, entertainment fee, and one year for clothing, heating, education and training, and automobiles.

## Conclusions

Our study revealed that the proportion of elderly with depressive symptoms was considerably high in 2013. Depressive symptoms were disproportionately concentrated in older adults with a lower income, favoring the rich. Most factors, such as income, living in rural areas, and social activity contributed to increasing the degree of pro-rich inequality. This pro-rich inequality was partially offset by residence in urban areas and working status. Possible policy and intervention strategies, such as narrowing the gap between the rich and the poor, encouraging the poor elderly to engage in all sorts of social activities, should be taken into account to reduce income-related inequalities.

## Additional file

Additional file 1: CES-D questions, English and Mandarin. (DOCX 17 kb)
