# Supplementary material for: Decomposing socioeconomic inequalities in depressive symptoms among the elderly in China
Source: BMC Public Health. 2016 Dec 1;16:1214. doi: 10.1186/s12889-016-3876-1 (PMC5134228; doi:10.1186/s12889-016-3876-1)
Supplement: Additional file 1: — CES-D questions, English and Mandarin. (DOCX 17 kb) [file 12889_2016_3876_MOESM1_ESM.docx]

**CES-D questions, English and Mandarin.**

**DC009.** I was bothered by things that don't usually bother me. 我因一些小事而烦恼。

1. Rarely or none of the time (<1 day) 很少或者根本没有（<1天）
2. Some or a little of the time (1-2 days) 不太多 （1-2天）
3. Occasionally or a moderate amount of the time (3-4 days) 有时或者说有一半的时间（3-4天）
4. Most or all of the time (5-7 days) 大多数的时间(5-7天)

**DC010.** I had trouble keeping my mind on what I was doing. 我在做事时很难集中精力。

1. Rarely or none of the time (<1 day) 很少或者根本没有（<１天）
2. Some or a little of the time (1-2 days) 不太多（１-２天）
3. Occasionally or a moderate amount of the time (3-4 days)　有时或者说有一半的时间（3-4天）
4. Most or all of the time (5-7 days) 大多数的时间（5-7天）

**DC011.** I felt depressed.我感到情绪低落。

1. Rarely or none of the time (<1 day) 很少或者根本没有（<１天）
2. Some or a little of the time (1-2 days) 不太多（１-２天）
3. Occasionally or a moderate amount of the time (3-4 days)　有时或者说有一半的时间（3-4天）
4. Most or all of the time (5-7 days) 大多数的时间（5-7天）

**DC012.** I felt everything I did was an effort.我觉得做任何事都很费劲。

1. Rarely or none of the time (<1 day) 很少或者根本没有（<１天）
2. Some or a little of the time (1-2 days) 不太多（１-２天）
3. Occasionally or a moderate amount of the time (3-4 days)　有时或者说有一半的时间（3-4天）
4. Most or all of the time (5-7 days) 大多数的时间（5-7天）

**DC013.** I felt hopeful about the future. 我对未来充满希望。

1. Rarely or none of the time (<1 day) 很少或者根本没有（<１天）
2. Some or a little of the time (1-2 days) 不太多（１-２天）
3. Occasionally or a moderate amount of the time (3-4 days)　有时或者说有一半的时间（3-4天）
4. Most or all of the time (5-7 days) 大多数的时间（5-7天）

**DC014.** I felt fearful. 我感到害怕。

1. Rarely or none of the time (<1 day) 很少或者根本没有（<１天）
2. Some or a little of the time (1-2 days) 不太多（１-２天）
3. Occasionally or a moderate amount of the time (3-4 days)　有时或者说有一半的时间（3-4天）
4. Most or all of the time (5-7 days) 大多数的时间（5-7天）

**DC015.**My sleep was restless.我的睡眠不好。

1. Rarely or none of the time (<1 day) 很少或者根本没有（<１天）
2. Some or a little of the time (1-2 days) 不太多（１-２天）
3. Occasionally or a moderate amount of the time (3-4 days)　有时或者说有一半的时间（3-4天）
4. Most or all of the time (5-7 days) 大多数的时间（5-7天）

**DC016.** I was happy. 我很愉快。

1. Rarely or none of the time (<1 day) 很少或者根本没有（<１天）
2. Some or a little of the time (1-2 days) 不太多（１-２天）
3. Occasionally or a moderate amount of the time (3-4 days)　有时或者说有一半的时间（3-4天）
4. Most or all of the time (5-7 days) 大多数的时间（5-7天）

**DC017.** I felt lonely. 我感到孤独。

1. Rarely or none of the time (<1 day) 很少或者根本没有（<１天）
2. Some or a little of the time (1-2 days) 不太多（１-２天）
3. Occasionally or a moderate amount of the time (3-4 days)　有时或者说有一半的时间（3-4天）
4. Most or all of the time (5-7 days) 大多数的时间（5-7天）

**DC018.** I could not get "going." 我觉得我无法继续我的生活。

1. Rarely or none of the time (<1 day) 很少或者根本没有（<１天）
2. Some or a little of the time (1-2 days) 不太多（１-２天）
3. Occasionally or a moderate amount of the time (3-4 days)　有时或者说有一半的时间（3-4天）
4. Most or all of the time (5-7 days) 大多数的时间（5-7天）
